# Effects of a Dietary Multienzyme Extract on Isotope Biokinetics in Red Claw Crayfish *Cherax quadricarinatus* Juveniles

**DOI:** 10.1155/2024/5538632

**Published:** 2024-11-15

**Authors:** Emily Sol García Martínez, Analía Verónica Fernández-Giménez, Laura Susana López Greco, Miquel Planas

**Affiliations:** ^1^Departamento de Biodiversidad y Biología Experimental, Facultad de Ciencias Exactas y Naturales, Universidad de Buenos Aires, Buenos Aires, Argentina; ^2^Instituto de Biodiversidad y Biología Experimental y Aplicada (IBBEA), CONICET-Universidad de Buenos Aires, Buenos Aires, Argentina; ^3^Instituto de Investigaciones Marinas y Costeras (IIMYC), CONICET-Facultad de Ciencias Exactas y Naturales, Universidad Nacional de Mar del Plata, Mar del Plata, Argentina; ^4^Laboratorio de Ecotoxicología de Invertebrados Acuáticos, Instituto Patagónico del Mar, Universidad Nacional de la Patagonia “San Juan Bosco” (IPaM-UNPSJB), Puerto Madryn, Argentina; ^5^Integrative Marine Ecology Group (INMARE), Department of Marine Ecology and Resources, Institute of Marine Research (IIM-CSIC), Vigo, Spain

**Keywords:** exogenous enzymes, fractionation, shrimp waste, stable isotopes, trophic discrimination factors, turnover

## Abstract

Understanding the nutritional and metabolic physiology of aquatic organisms is essential for optimizing aquaculture practices and informing ecological models. We investigated the influence of dietary composition, specifically the incorporation of multienzyme extract derived from *Pleoticus muelleri* waste, on the growth and metabolic processes of juvenile *Cherax quadricarinatus*. We investigated how these dietary changes influence dietary assimilation and tissue turnover using stable isotope δ^13^C and δ^15^N dynamics, in both the pleon muscle and hepatopancreas. Juvenile crayfish were subjected to two isocaloric diets for a 90-day period: a control diet (diet C) and a diet supplemented with a multienzyme extract (diet E) from red shrimp waste. Despite comparable growth rates, isotopic analysis (δ^13^C and δ^15^N) of the pleon muscle and hepatopancreas revealed distinct metabolic patterns between both dietary treatments. The hepatopancreas exhibited accelerated isotopic turnover relative to muscle tissue, irrespective of diet, suggesting a more dynamic metabolic role. Diet E further accelerated turnover rates in both tissues, indicative of enhanced nutrient assimilation and utilization. Consistent isotopic disparities between the hepatopancreas and muscle tissues highlighted tissue-specific metabolic functions, with the hepatopancreas serving as a metabolic hub. Molting-induced shifts in isotopic patterns underscored the dynamic interplay between metabolic processes and nutrient mobilization. Isotopic equilibrium was reached earlier for δ^13^C than δ^15^N, with lower discrimination factors in the hepatopancreas. While δ^13^C primarily supported metabolic processes, δ^15^N contributed substantially to growth, especially in muscle. These findings illuminate the complex interplay of dietary composition, isotopic fractionation, and physiological regulation in *C. quadricarinatus*. The metabolic enhancements induced by the diet supplemented with the extract warrant further investigation to optimize nutrient utilization and growth performance in aquaculture settings.

**Summary**

• This is the first isotopic study in crayfish *Cherax quadricarinatus* juveniles.
• This study provides the first trophic discrimination factor (TDF) estimate for the species.
• This study shows enhanced digestion and diet assimilation with multienzyme extract supplementation.
• This study shows isotopic trends influenced by molting.

## 1. Introduction

Modern aquaculture plays a crucial role in the global food system, characterized by rapid production growth and the adoption of innovative feed ingredients, production technologies, farm management practices, and value chains [1]. Despite this progress, fish and crustacean farming remain heavily reliant on fishmeal sourced from fisheries, a dependency that exerts significant pressure on marine ecosystems and contributes to the depletion of wild fish stocks [2]. Additionally, the high costs associated with fishmeal production drive up operational expenses in aquaculture [3]. As a result, a major challenge for the industry is the development of alternative feed ingredients and functional additives that not only enhance productivity but also support economic viability with environmental sustainability.

A promising strategy gaining attraction in aquaculture nutrition is the inclusion of exogenous enzymes as dietary supplements [4]. These enzymes improve the digestibility of less digestible ingredients, such as alternative animal protein sourced (e.g., poultry by-products and insect meals), as well as plant, algae, or fungi-based proteins. This reduces reliance on fishmeal, promotes more efficient feed conversion and energy utilization, and correlates with a reduction in nutrient waste in aquaculture systems [5–7].

The high cost of commercially available exogenous enzymes represents a significant challenge to their widespread adoption in aquaculture. To address this problem, researchers are exploring cost-effective alternatives like multienzyme extracts derived from marine fishery by-products. This approach promotes circular economy principles by valorizing waste streams and creating job opportunities associated with the development and production of these multienzyme extracts [7–10].

While research on the benefits of exogenous enzymes in aquaculture has been extensive, particularly for fish such as rainbow trout (*Oncorhynchus mykiss*) [5, 7, 11–13], studies on crustaceans, such as the shrimp *Litopenaeus vannamei*, remain limited [5, 14, 15]. The impact of these exogenous enzymes on other commercially important decapod crustaceans, such as *Cherax quadricarinatus*—a globally cultivated species prized for both food and ornamental purposes—is even less understood [16, 17]. *C. quadricarinatus* possesses attractive attributes such as eurythermal nature, fast growth, and adaptability to various conditions, making it a preferred choice for commercial aquaculture [17–19].

Although *C. quadricarinatus* is equipped with a well-developed digestive system with diverse digestive enzymes, incorporating exogenous enzymes into the diet could enhance digestion and potentially boost productivity in aquaculture conditions. This would allow the use of new or locally available, less digestible feed ingredients, thereby improving profitability in ex situ culture [20]. Despite these potential benefits, research on the use of exogenous enzymes for redclaw crayfish is still in its early stages, highlighting a significant gap in our understanding of how to optimize aquaculture practices for this species. Further research is needed to advance our knowledge and promote sustainable growth within the redclaw crayfish aquaculture sector.

The assimilation efficiency of experimental diets can be evaluated through stable isotope analysis (SIA), focusing on δ^13^C and δ^15^N. Tissue isotopic composition reflects dietary intake, incorporating an enrichment factor known as the trophic discrimination factor (TDF) [21–25]. Consequently, SIA is a valuable tool for investigating the incorporation and turnover of dietary resources within tissues (biokinetics) [26–29]. While δ^13^C values provide insights into dietary source and assimilation, δ^15^N indicates the species's trophic position [30]. Following a dietary shift, tissue δ^13^C and δ^15^N values gradually change until reaching equilibrium with the diet, as lighter isotopes (^12^C and ^14^N) are preferentially utilized in metabolic processes [22, 23]. Modeling these isotopic changes offers valuable information on the physiological and trophic characteristics of the species [31–33].

Previous stable isotope studies on some *Cherax* species have estimated turnover rates and TDFs for δ^13^C and δ^15^N, revealing variability influenced by tissue type, diet, and specimen age, as well as significant interindividual isotopic variation [34–37]. However, the impact of diets supplemented with a multienzyme extract on isotope biokinetics and tissue–diet discrimination factors within *Cherax* species remains unexplored.

Rodríguez et al. [38] investigated the potential of exogenous enzymes as dietary supplements for redclaw crayfish. In vitro assays suggested that multienzyme extracts derived from various fishery by-products (*Pleoticus muelleri*, *Artemesia longinaris*, and *Ilex argentinus*) could enhance digestion in juvenile *C. quadricarinatus*. Corroborating these findings, a recent study reported increased lipase activity in juveniles fed a diet enriched with multienzyme extracts sourced from shrimp discards [39]. These juveniles exhibited elevated hepatopancreatic lipid content and increased pleon glycogen, while protein levels decreased in both tissues. Given the unexplored influence of multienzyme extract supplementation on isotope biokinetics and tissue-diet discrimination factors in *Cherax* species, this study aims to examine how such supplementation interacts with endogenous digestive enzymes, potentially augmenting nutrient assimilation.

We hypothesize that multienzyme extracts will enhance digestive efficiency, thereby facilitating nutrient assimilation and inducing observable changes in isotope profiles and tissue turnover rates. The potential digestive benefits of exogenous enzymes derived from shrimp discards could establish a foundation for improving the digestibility of various diets, contributing to environmental sustainability through protein waste recycling. Furthermore, understanding the biology of *C. quadricarinatus* is paramount for both conservation initiatives and aquaculture practices, enabling sustainable population and ecosystem management, as well as optimized ex situ production.

## 2. Materials and Methods

### 2.1. Animals

Juveniles of *C. quadricarinatus* (von Martens, 1868) were obtained from five ovigerous females under controlled laboratory conditions. These females originated from our reproductive stocks at the Facultad de Ciencias Exactas y Naturales, Universidad de Buenos Aires (Argentina). Each female was housed individually in a glass aquaria (60 × 40 × 30 cm) filled with dechlorinated water and a polyvinyl chloride (PVC) tube (20 cm in length, 7.5 cm in diameter) as shelter. Water quality was maintained through continuous aeration and specific parameters: pH 7–8, hardness 70–100 mg/L as CaCO_3_ equivalents, and oxygen concentration exceeding 5 mg/L. A 14-h light and 10-h dark photoperiod was implemented to mimic natural daylight, while water temperature was kept constant at 27 ± 1°C [40, 41]. Females were fed ad libitum daily with a balanced diet consisting of *Elodea* sp. and a commercial balanced food for tropical fish (Tetracolor, TETRA), containing 47.5% protein, 6.5% lipids, 2.0% fiber, and 6.0% moisture. The diet has proven effective for optimal growth and reproduction in redclaw crayfish, as confirmed by previous studies [42].

The development stages of eggs across all ovigerous females were carefully monitored to ensure uniformity. This approach resulted in consistent juvenile characteristics, including similar size at hatching. Upon reaching the independent juvenile stage III (~15–17 mg), juveniles were transferred to glass aquaria equipped with large plastic nets and PVC tubes (3 cm in length, 1.5 cm in diameter) to serve as shelters. The enriched environment, featuring multiple shelters and nets, effectively minimizes aggressive interactions and reduced cannibalism. Stocking density was maintained at of 50–55 juveniles/m^2^. For 1.5 months, juveniles were kept under the same controlled conditions as the broodstock, including temperature, water quality, photoperiod, and feeding regime. Additionally, the aquaria were weekly monitored to identify individuals preparing to molt and to redistribute juveniles by size, ensuring more equitable competition. Under these conditions, we estimate that cannibalism was below 20% (based on unpublished data from our laboratory).

At the start of the experiment, only intermolt juvenile crayfish with a similar weight (1.46 ± 0.20 g, range: 1.2–1.8 g; 4.05 ± 0.23 cm total length, range: 3.5–4.5 cm; and 1.95 ± 0.12 cm carapace length, range 1.8–2.2 cm) were randomly selected and transferred to the designated experimental units.

### 2.2. Experimental Diets

The multienzyme extract utilized in this study was derived from Argentine red shrimp *Pleoticus muelleri* (Solenoceridae) waste. Frozen waste samples were transported to the laboratory, thawed, and processed by removing and homogenizing the cephalothorax. Approximately 1 kg of waste was crushed in ice-cold distilled water (1:2 w/v). The resulting material was centrifuged at 10,000 *g* for 30 min at 4°C (Presvac EPF 12R), and the supernatant, containing the multienzyme extract, was frozen at −20°C. The enzymatic activity of the shrimp extract, including acid peptidase activity (pH6), alkaline peptidase activity (pH8), and soluble protein concentrations, were determined according to Rodríguez et al. [38]. Lipase activity was measured at pH 8 based on Nolasco-Soria et al. [43].

A modified omnivorous diet was formulated for this study, based on Rodríguez et al. [7]. The control diet (C) comprised fish meal (25%), shrimp meal (10%), soybean meal (35%), wheat flour (20%), and rice flour (10%). The experimental diet (E) incorporated a specific volume of multienzyme extract to achieve final concentrations of 38 U/100 g alkaline peptidase, 35 U/100 g acid peptidase, and 292 U/100 g lipase, as recommended in a previous study [39]. Both diets were oven-dried at 40°C for 7 h.

The proximal biochemical composition of diet E (25.5% protein, 15.1% lipids, 37.4% carbohydrates, 8.5% ash, and 13.5% moisture) did not significantly differ from that of diet C (24.1% protein, 14.1% lipids, 36.4% carbohydrates, 7.9% ash, and 17.5% moisture). The protein and lipid content of both diets aligns with ranges reported as suitable for redclaw crayfish in previous studies [44, 45].

### 2.3. Experimental Design and Sample Collection

This study involved 52 redclaw crayfish juveniles with similar weight (1.46 ± 0.20 g; 4.05 ± 0.23 cm total length). The juveniles were randomly assigned to one of two treatment groups: a control diet (C) or a diet supplemented with multienzyme extract (E), resulting in 24 replicates for group C and 28 for group E. Each crayfish was individually housed in a 12 × 7 × 14 cm plastic aquarium containing 2 L of dechlorinated water, with continuous aeration and a PVC tube shelter. The experimental environment maintained a constant temperature of 27 ± 1°C and a 14 L:10D photoperiod. Water quality was maintained by complete water changes three times weekly, removing uneaten food and waste. A daily morning feeding regimen provided crayfish with 3% of their body weight in food, while mortality and molting were recorded daily. Postmolt, crayfish weight was determined to adjust daily feed rations accordingly.

At intervals of 15, 30, 45, 60, 75, and 90 days, three replicates from each treatment were randomly selected, weighted (± 0.1 mg), and euthanized using ice anesthesia for 5 min. Additionally, at the onset of the experiment (day 0), three juveniles with similar initial weights were sacrificed to establish a baseline status before diet initiation. Hepatopancreas and pleon tissues from sampled juveniles were excised and lyophilized for subsequent SIA of δ^13^C and δ^15^N, as well as total carbon and nitrogen. Three feed samples from each experimental diet were also collected at various time points throughout the experiment and subjected to SIA.

A concurrent trial assessed molting in 62 juveniles, 31 replicates per treatment C and E (1.44 ± 0.19 g, 4.03 ± 0.18 cm total length, and 1.89 ± 0.1 cm carapace length) under identical experimental conditions. The experiment lasted 90 days, as this period allows juveniles of this size to undergo two or three molts, providing a reliable indication of the multienzyme extract's effects on metabolism and/or growth. During the trial, daily mortality and molting were monitored. On day 45, 15 individuals from each treatment group were randomly chosen; their body mass and size were recorded and then were sacrificed. The remaining juveniles were subjected to the same procedure at the end of the experiment on day 90. Further details were provided in Martínez et al. [39].

### 2.4. SIA

Muscle (pleon) and digestive gland (hepatopancreas) dried samples from each crayfish were treated with 1% hydrogen chloride (HCl) until carbon dioxide (CO_2_) release ceased, ensuring complete removal of any exoskeleton remnants [46, 47]. Excess HCl was then removed without rinsing to minimize dissolved organic matter loss [48], followed by a redrying at 60°C.

Dried samples of the muscle, the hepatopancreas, and diets were ground into a fine powder and divided into two portions. One portion remained untreated, while lipids were removed from the other using chloroform:methanol (2:1) [49]. Samples were mixed for 2 min, rested for 30 min, centrifuged (10 min at 3400 rpm), and supernatants discarded. This lipid extraction process was repeated thrice, followed by pellet redrying at 60°C for 24 h to eliminate residual solvent. Notably, experimental feeds were not subjected to lipid removal as crayfish can assimilate dietary lipids [50].

δ^13^C, δ^15^N, and elemental composition analyses (total C and total N percentage) were conducted at Servizos de Apoio á Investigación (SAI) of the University of A Coruña (Spain). Samples were analyzed using continuous-flow isotope ratio mass spectrometry using a FlashEA1112 elemental analyzer (Thermo Finnigan, Italy) coupled to a Delta Plus mass spectrometer (FinniganMat, Bremen, Germany) through a Conflo II interface. Carbon and nitrogen stable isotope abundance was expressed as permil (‰) relative to Vienna Pee Dee Belemnite (VPDB) and atmospheric air, according to the following equation:

  $$\begin{array}{c} \delta X\;=\left(\frac{R_{\text{sample }}}{R_{\text{reference}}}\right)-1, \end{array}$$

where *X* is ^13^C or ^15^N and *R* is the corresponding ^13^C/^12^C or ^15^N/^14^N ratio. International reference materials were used for δ^13^C USGS 40, −26.39‰; USGS 41a, +36.55‰; NBS 22, −30.031‰; and USGS 24, −16.049‰) and δ^15^N (USGS 40, −4.52‰; USGS 41a, +47.55‰; IAEA-N−1, +0.4‰; and IAEA-N-2, +20.3‰); USGS−25, −30.4‰). The precision (standard deviation) for the analysis of δ^13^C and δ^15^N in the laboratory standard (acetanilide) was ± 0.15‰ (1-sigma, *n* = 10). Standards were run every 10 biological samples. The precision (standard deviation) for the analysis of δ^13^C and δ^15^N of the laboratory standard (acetanilide) was ± 0.15‰ (1-sigma, *n* = 10). Standards were run every 10 biological samples. The isotopic analysis procedure fulfilled the requirements of the International Organization for Standardization (ISO) 9001 standard. The laboratory is submitted to annual intercalibration exercises (e.g., forensic isotope ratio mass spectrometry scheme [FIRMS], LGC Standards, UK).

### 2.5. Data Treatment

Changes in δ^13^C and δ^15^N from day 0 to day 90 were modeled using a first-order one-compartment model [51], as a function of developmental progress (days) [29, 52]. The empirical equation describing isotopic changes with growth is as follows [53]:

  $$\begin{array}{c} \delta =\delta_{\text{eq }}+ae^{-\left(m+k\right)t}, \end{array}$$

where *δ* is the isotopic (δ^13^C or δ^15^N) value at day *t*, *δ*_eq_ is the model-fitted δ^15^N or δ^13^C isotopic ratio in equilibrium with the diet, *a* is the difference between the initial isotopic value (day 0) and the equilibrium isotopic value (*a* = δ_i_–δ_eq_), *t* is the time (days), *m* is the model-fitted metabolic constant, and *k* is the growth rate parameter calculated for each replicate considering dry weight changes from day 0 to the sampling day. The growth rates *k* were calculated at each sampling day as follows:

  $$\begin{array}{c} k=\ln\left(W_{t}/W_{i}\right)/t, \end{array}$$

where *W*_t_ is the weight attained at time *t* and *W*_i_ is the initial weight.

Consistent with other studies, we assumed independent interactions between growth and metabolism, despite acknowledging their recognized covariation with body size [33, 54].

To determine half-life (D_50_ or G_50_) or equilibrium (D_95_ or G_95_) tissue turnover, the empirical equation was solved for *α* = 50% or 95%, respectively. The x-fold increase in dry weight (G_α_) and the days (D_α_) required to achieve a specific tissue turnover percentage were calculated as follows [31]:

  $$\begin{array}{c} {\text{D}}_{\alpha }=\ln\left(1-\alpha /100\right)/\left(m+k\right) \end{array}$$

Values for *k* and D_α_ were determined for each sampling day and experimental group. Subsequently, D_α_ values were converted to G_α_ values using corresponding equations relating time and weight.

The relative contribution of tissue turnover derived from growth (P_g_) and metabolism (P_m_) was calculated as follows:

  $$\begin{array}{c} {\text{P}}_{\text{g}}=2\left(G_{50}-1\right)/G_{50}, \end{array}$$

  $$\begin{array}{c} {\text{P}}_{\text{m = }}\left(2-G_{50}\right)/G_{50}. \end{array}$$

Tissue–diet discrimination factor (Δδ) for δ^13^C and δ^15^N were calculated as the difference between equilibrium fish tissue and diet isotope values (*Δδ* = *δX*_eq_ − *δX*_diet_) [51].

### 2.6. Statistical Analysis

All statistical analyses were performed using R Studio version 4.1.2 [55]. Values are presented as mean ± standard deviation, with a significance level of 0.05. For variables following a normal distribution, data normality and heteroscedasticity were assessed using the Shapiro–Wilk and Levene's tests, respectively. Two-way analysis of variances (ANOVAs) were applied to compare isotope data means, with Tukey's honestly significant difference (HSD) post hoc test applied for significant differences. For survival analysis, a generalized linear model with a binomial distribution was applied, with “diet” as a fixed factor. Similarly, the number of molts at day 90 was analyzed using a generalized linear model with a Poisson distribution, incorporating “diet” as a fixed factor.

The stepAIC function in the MASS R package evaluated the lipid effect (C:N ratio) on δ^13^C and δ^15^N in crayfish tissues (pleon muscle and hepatopancreas). Consequently, equations were developed to mathematically convert isotope values between bulk and lipid-free crayfish tissues. Paired *t*-tests examined differences in stable isotopes between these tissue types. The empirical equation model proposed by Hesslein, Hallard, and Ramlal [53] was fitted to isotope data using Statistica 8.0 (StatSoft, USA).

## 3. Results

### 3.1. Growth Performance and Molts

**Figure 1:**
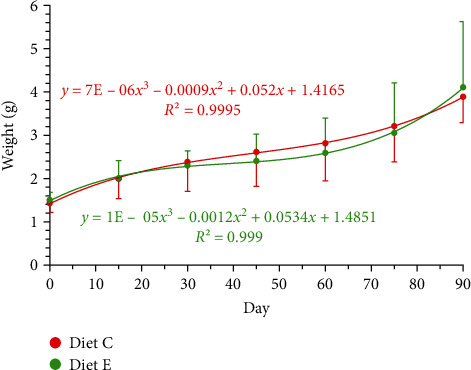
Wet weight (g) in *Cherax quadricarinatus* juveniles fed on two different diets (C, control diet; E, enzyme extract diet) for 90 days.

The overall survival of the experiment was 83% (*χ*^2^ = 1.002; df = 1; *p* = 0.317). Initial wet weights of juveniles fed diets C and E (1.43 ± 0.21 g and 1.50 ± 0.18 g, respectively) showed no significant difference (*t*-value = −1.145; df = 34; *p* = 0.581). Weight gain was comparable between dietary treatments, with final values at day 90 (3.89 ± 0.60 g and 4.11 ± 1.50 g, respectively) also exhibiting no significant difference (*t*-value = −0.236; df = 4; *p* = 0.271) (Figure 1). The number of molts at the end of the experiment was similar between treatments (*χ*^2^ = 0.249; df = 1; *p* = 0.618), with an average of 2.7 ± 0.82 molts per animal. This finding aligns with the results on growth and molting of the concurrent experiment, where the number of molts was also not significantly affected by diet, with an average of two molts per animal throughout the study [39].

### 3.2. Effect of Lipids on Stable Isotopes

**Figure 2:**
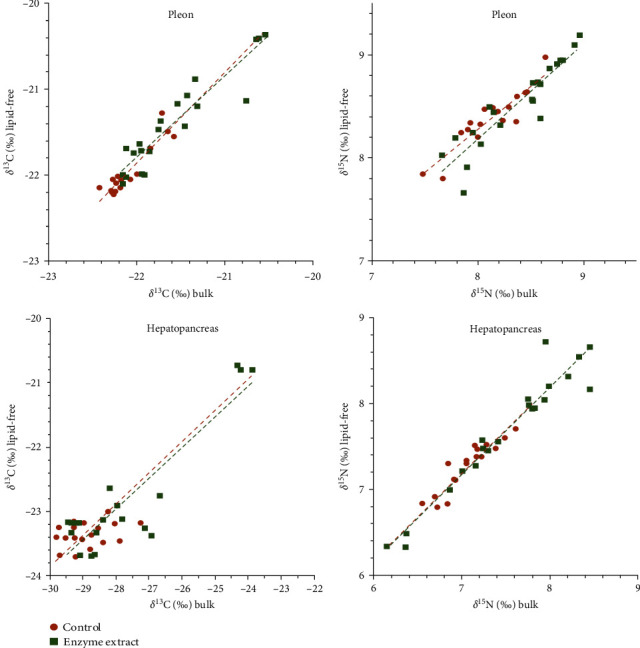
Relationships for δ^13^C and δ^15^N between bulk and lipid-free tissues (muscle and hepatopancreas) in *Cherax quadricarinatus* juveniles fed on two different diets (C, control diet; E, multienzyme extract diet) for 90 days.

As both dietary treatments exhibited similar lipid extraction profiles, isotope data from crayfish juveniles were pooled for analysis (Figure 2). Lipid extraction significantly affected δ^13^C (paired *t*-test; muscle: *n* = 38, *t* = 6.49, *p* < 0.001; hepatopancreas: *n* = 39, *t* = 31.72, *p* < 0.001) and δ^15^N values (paired *t*-test; muscle: *n* = 38, *t* = 8.10, *p* < 0.001; hepatopancreas: *n* = 39, *t* = 10.46, *p* < 0.001) in crayfish tissues. However, the average isotopic change in pleon muscle postlipid extraction was minor, with increases of only + 0.17 ± 0.16‰ in δ^13^C (0.8%) and + 0.20 ± 0.15‰ in δ^15^N (2.5%). A similar small effect was observed for δ^15^N in the hepatopancreas (+0.18 ± 0.15‰; 2.4% increase), but δ^13^C in lipid-free hepatopancreas exhibited a substantial decrease of −5.7 ± 1.01‰ (18.1%). Equations for converting isotope values from bulk to lipid-free tissues are presented in Table 1.

While the initial regression model for δ^13^C in the hepatopancreas was significant, caution is advised due to the influence of three outlier values at day 0 (Figure 2). Excluding these outliers significantly reduced the model's *R*^2^ to 0.045.

### 3.3. Diets

Bulk diets did not differ in δ^13^C values (F_1,4_ = 0.026; *p* = 0.881) or δ^15^N (F_1,4_ = 1.233; *p* = 0.329). Mean isotope values were −26.8 ± 0.1 ‰ for δ^13^C in both diets and 7.1 ± 0.3 ‰ for δ^15^N in diet C and 7.3 ± 0.1 ‰ in diet E (Table 2). The ratios total C:total N were also similar in both diets (9.7) (F_1,4_ = 1.079; *p* = 0.358).

Lipid-free diets similarly showed no significant differences in δ^13^C (F_1,4_ = 0.425; *p* = 0.103) or δ^15^N (F_1,4_ = 3.428; *p* = 0.138) values between experimental diets. Mean δ^1^³C values were −25.4 ± 0.3‰ in diet C and −25.0 ± 0.2‰ in diet E, while mean δ^15^N values were 6.7 ± 0.6‰ in diet C and 7.4 ± 0.4‰ in diet E (Table 2). C:N values also remained similar between lipid-free diets (7.4) (F_1,4_ = 0.364; *p* = 0.579).

### 3.4. Crayfish Juveniles

Initial isotope values differed significantly between lipid-free pleon muscle and hepatopancreas (F_1,8_ = 477.4; *p* < 0.001 for δ^13^C; F_1,8_ = 758.1; *p* < 0.001 for δ^15^N), with the muscle exhibiting higher values (Table 2). The hepatopancreas also displayed a nearly twofold higher C:N ratio compared to pleon muscle (F_1,8_ = 42.25; *p* < 0.001), indicating greater lipid content.

**Figure 3:**
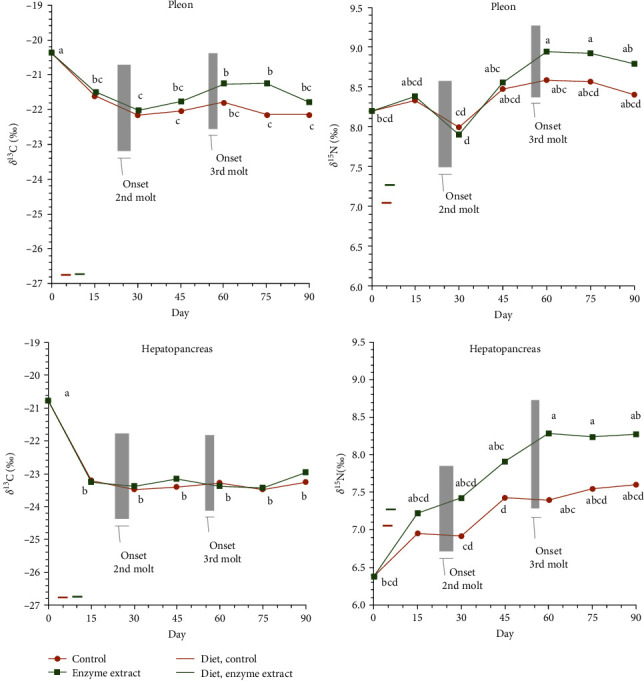
Changes with time of δ^13^C and δ^15^N values in the muscle and hepatopancreas of *Cherax quadricarinatus* juveniles (C, control diet; E, enzyme extract diet) for 90 days. Standard deviations are not provided for clarity (*n* = 3). The onset of the 2nd and 3rd molts is indicated. Colored horizontal lines indicate the isotope values in the experimental diets C and E. Different letters indicate statistical significance.

Over time, δ^13^C values rapidly decreased toward dietary levels, especially in the hepatopancreas (Figure 3). Both diets showed similar trends, particularly evident in the hepatopancreas, with no significant diet–day interaction. However, δ^13^C values in the muscle varied across both treatments (F_1,6_ = 18.704; *p* < 0.001) and days (F_1,6_ = 33.445; *p* < 0.001). The hepatopancreas δ^13^C changes were solely influenced by time (F_1,6_ = 99.972; *p* < 0.001).

**Figure 4:**
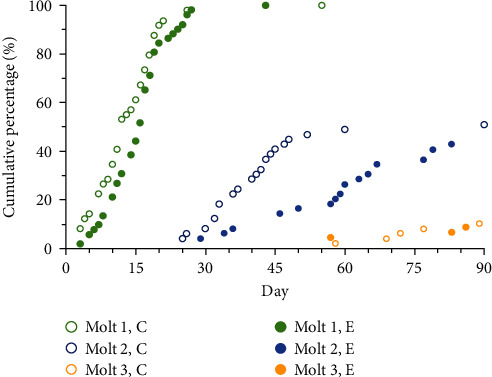
Evolution of molting stages in *Cherax quadricarinatus* juveniles fed on two different diets (C, control diet; E, multienzyme extract diet) for 90 days. Data are presented as the cumulative percentage (%) of individuals molted over time.

The dietary regime exerted a stronger influence on δ^15^N value progression compared to δ^13^C, although changes were not uniform, especially in the pleon muscle (Figure 3). Muscle δ^15^N values differed between treatments (F_1,6_ = 7.331; *p* = 0.011) and days (F_1,6_ = 12.572; *p* < 0.001), with a notable depletion observed by day 30, following the second molt (Figure 4). Hepatopancreas δ^15^N values were also significantly influenced by both treatment (F_1,6_ = 47.449; *p* < 0.001) and day (F_1,6_ = 35.580; *p* < 0.001).

### 3.5. Turnover Rates

**Figure 5:**
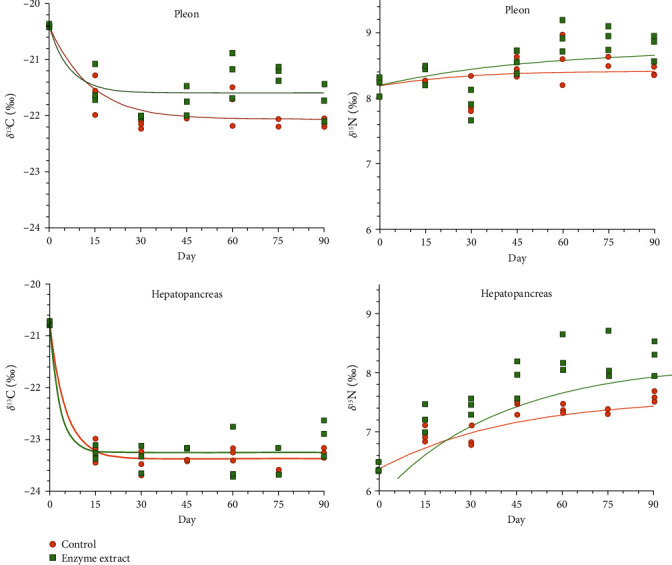
Progression of δ^13^C and δ^15^N values with time (days) in the muscle and hepatopancreas of *Cherax quadricarinatus* juveniles (C, control diet; E, enzyme extract diet).

Fitting isotopic values to the time-based model revealed rapid turnover rates for δ^13^C, especially in the hepatopancreas (Figure 5 and Table 3). Muscle metabolic turnover rates (m) were 0.077 and 0.192 day^−1^ for diets C and E, respectively, while hepatopancreas rates were higher (0.153 and 0.304 day^−1^, respectively). Notably, half-life (D_50_) and near-complete turnover (D_95_) estimates were nearly halved with diet E. G_50_ values for both diets and tissues were similar, ranging from 1.01 to 1.13.

δ^15^N turnover rates were considerably slower than those for δ^13^C, regardless of diet or tissue type (Table 3). Muscle metabolic turnover rates (m) were 0.033 and 0.020 day^−1^ for diets C and E, respectively, while hepatopancreas rates were 0.023 and 0.025 day^−1^, respectively. Muscle δ^15^N half-life (D_50_) ranged from 15.8 to 23.4 days, corresponding to G_50_ values of 1.33–1.51. Hepatopancreas D_50_ and G_50_ values were similar at 20.2–20.4 and 1.44–1.45, respectively.

Isotopic equilibrium (δ^13^C_eq_ and δ^15^N_eq_) was attained in less than 1 month for δ^13^C and near the end of the experiment for δ^15^N. While δ^13^C_eq_ values were similar between diets, they were slightly lower (more negative) in the hepatopancreas. Conversely, δ^15^N_eq_ values were slightly higher in diet E and pleon muscle (Figure 5 and Table 3). Caution is advised when interpreting δ^15^N_eq_ in the pleon muscle due to the low *R*^2^ value.

### 3.6. Isotope Fractionation

Estimated discrimination factors (Δδ) (Table 3) for δ^13^C in bulk diets were lower in the hepatopancreas (3.3‰ and 3.4‰ for treatments C and E, respectively) compared to muscle (4.7‰ and 5.1‰, respectively). Conversely, discrimination factors for δ^15^N ranged from 1.4‰ to 1.6‰ in muscle and from 0.5‰ to 1.1‰ in the hepatopancreas. Notably, lipid extraction significantly reduced δ^13^C discrimination factors (Δδ′) compared to bulk diet estimates but had a minimal effect on δ^15^N discrimination factors, except for a slight decrease in the hepatopancreas for diet C.

### 3.7. Contribution to Growth and Metabolism

**Figure 6:**
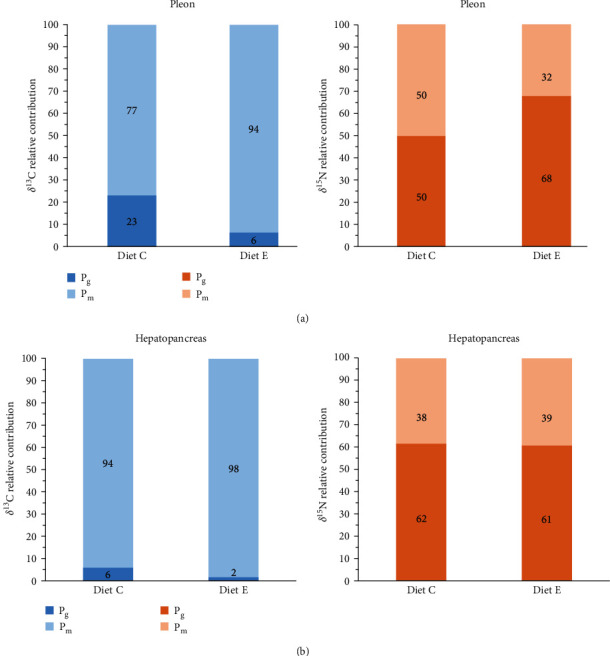
Relative contribution of growth (P_g_) and metabolic (P_m_) turnovers to isotopic changes (δ^13^C and δ^15^N) in the pleon muscle (a) and hepatopancreas (b) of *Cherax quadricarinatus* juveniles fed on two different diets (C, control diet; E, multienzymatic extract) diet for 90 days.

Estimates of the relative contributions of growth (P_g_) and metabolic (P_m_) turnover to isotopic changes indicated that δ^13^C primarily fueled metabolism in both diets C (77% in muscle and 94% in hepatopancreas) and E (94% in muscle and 98% in hepatopancreas) (Figure 6). While ^13^C contributed more to muscle growth (6%–23%) than hepatopancreas growth (2%–6%), ^15^N contributed substantially more to growth (38%–50% in diet C and 32%–39% in diet E), especially in pleon muscle for diet C (50%).

## 4. Discussion

Previous research [50] linked the carbon-to-nitrogen (C:N) ratio of food sources to protein content, with ratios exceeding 6 indicating lower protein levels [56]. Both experimental diets exhibited high C:N ratios (~9.7), suggesting lower protein content (around 24%–25%). Nevertheless, this protein content falls within the range supporting optimal growth in juveniles *C. quadricarinatus* [44].

Enzyme supplements have the potential to improve feed efficiency and other aspects of crustacean aquaculture by enhancing nutrient digestion and absorption, reducing feed costs and improving growth and survival. Adding a multienzyme extract derived from *P. muelleri* shrimp waste to the diet at a specific level (38 U/100 g of alkaline peptidase, 35 U/100 g of acid peptidase, and 292 U/100 g of lipase) seemed to influence nutrient digestion and absorption by modulating the activity of digestive enzymes. Specifically, the multienzyme extract increased the specific activity of endogenous digestive peptidases and exhibited a trend of rising lipase activity in *C. quadricarinatus* juveniles, as demonstrated by recent results [39].

### 4.1. Effect of Lipids on SIA

Stable isotopes are commonly analyzed in fat-free tissues when studying food webs and feeding habits [57] to ensure data accuracy. Removing fats from muscle tissue in our study led to minimal isotope value increases (+0.2 ± 0.2‰; 0.8% and 2.5% for δ^13^C and δ^15^N, respectively). Conversely, hepatopancreas lipid removal caused a substantial δ^13^C increase (18.4%; +5.2 ± 0.9‰), while δ^15^N values remained relatively stable (+0.2 ± 0.2‰; 2.5%). Despite the statistical significance of these changes, we recommend removing fats before isotope analysis, especially in lipid-rich tissues like crayfish hepatopancreas, as suggested by previous decapod research [58].

### 4.2. Tissue Turnover and Isotope Fractionation

Both diets exhibited similar nutrient profiles (protein, carbohydrates, and lipids), as evidenced by comparable chemical composition, carbon-to-nitrogen ratios, and isotope values, suggesting similar nutritional value for crayfish. The primary anticipated difference in nutrient utilization stemmed from the multienzyme extract in one diet.

Overall, both δ^13^C and δ^15^N values were consistently lower in the hepatopancreas compared to muscle tissue across both diets, indicating significant intertissue fractionation [27]. While δ^13^C values equilibrated with diets faster than δ^15^N values in both tissues and diets, the rate of isotopic change (reflecting dietary element incorporation) was quicker for δ^13^C in the digestive gland. Distinct δ^15^N patterns emerged between diets.

Both diets yielded similar isotope values in crayfish throughout the experiment. Notably, crayfish rapidly incorporated dietary elements into their tissues, particularly for carbon (δ^13^C), reaching isotopic equilibrium within a month (half-life ≤ 8 days). However, δ^13^C value changes varied by tissue type and diet, with hepatopancreas values shifting twice as fast as muscle tissue. Crayfish fed diet E exhibited faster δ^13^C value changes than those fed diet C, suggesting lower metabolic activity in the latter. This implies the multienzyme extract potentially enhanced digestion and assimilation, aligning with a previous study reporting increased proteinase activity in extract-fed juveniles [39]. The extract boosted digestive enzyme activity, influencing energy storage (increased hepatopancreatic fat and muscle glycogen) without affecting overall growth. These findings, coupled with the faster isotope turnover in diet E, suggest a higher metabolic rate and improved nutrient digestion/absorption.

Crayfish juveniles undergo sequential molting stages, shedding their exoskeleton and rebuilding it while maintaining relatively constant body weight [59, 60]. Actual growth occurs during intermolt periods, utilizing nutrients from food to replace water absorbed during molting [61]. Weight gain depends on individual age and nutritional status [62], and these growth patterns are reflected in isotope values. Isotopic changes are closely linked to metabolic variations and nutrient mobilization across different molt stages.

Muscle tissue δ^15^N values dropped significantly around day 30, with a smaller δ^13^C decrease, coinciding with the second molt (days 20–28). A similar, less pronounced pattern occurred around day 60, aligning with the third molt onset. This pattern aligns with research on adult crayfish (*C. destructor*) by Carolan et al. [35], who observed muscle tissue nutrient loss during sexual maturation. While our crayfish were immature, nutrient mobilization during molting likely caused these isotope changes. Protein and lipid depletion postmolt, as observed in juvenile lobsters (*Thenus australiesis*) [60], might explain these patterns.

Decapod molting involves substantial enzymatic activity changes [63]. Digestive enzyme activity (chitinase, amylase, and protease) typically decreases before molting and increases afterward [64–66]. While hepatopancreatic digestive enzyme activity in *Callinectes arcuatus* decreases during molting, amylase and lipase activity increases [67]. These enzyme fluctuations significantly influence free amino acid availability [68], which declines markedly during molting due to reduced feeding and protease activity [69, 70]. Muscle shrinkage facilitating molting may be linked to these changes [66, 71]. Consequently, the observed pleon muscle δ^15^N declines in *C. quadricarinatus* around the second and third molts align with these digestive enzyme and physiological changes associated with molting.

Under controlled laboratory conditions with a single diet, animal tissues eventually reflect dietary isotope signatures. However, the rate of isotopic incorporation varies among organisms and tissues, influenced by protein intake, turnover, growth rate, and body mass. Additionally, within-animal tissue isotope incorporation rates differ due to varying protein utilization and turnover [72]. Both the pleon muscle and hepatopancreas reached isotopic equilibrium after ~4.3 half-lives in both diets, aligning with Hobson and Clark [26] findings of equilibrium attainment after four to five half-lives. Our half-life, equilibrium, and discrimination factor results align with those from *C. destructor* studies (Table 4).

Juveniles redclaw crayfish efficiently incorporated dietary elements into their tissues, as reflected by rapid isotope shifts. This rapid incorporation suggests a high protein turnover rate, especially in the hepatopancreas compared to muscle. These findings align with research on other shrimp and prawn species like *Penaeus esculentus* and *Macrobrachium borellii* [73, 74], where splanchnic tissues exhibit faster isotopic turnover than structural tissues [25].

Metabolic turnover rate (*m*) estimates an animal's body mass replacement independent of growth [53]. Muscle tissue δ^13^C values indicated a higher metabolic rate in crayfish fed diet E compared to diet C, suggesting more efficient energy use and faster equilibrium with the diet. While δ^15^N metabolic turnover in the muscle was lower, both the muscle and hepatopancreas showed higher metabolic contributions from diet C, implying increased protein catabolism. The hepatopancreas exhibited lower δ^13^C and δ^15^N enrichment than muscle, reflecting tissue-specific biosynthesis and amino acid composition differences. Its high metabolic contribution (P_m_ = 94%–98%) for δ^13^C underscores its central metabolic role, consistent across diets. Muscle P_m_ from diet E was similar to hepatopancreas but decreased to 77% in diet C.

Animal feces are typically enriched in heavier ^13^C and ^15^N isotopes due to digestive fractionation. Consequently, assimilated food exhibits lower isotopic enrichment compared to feces [35]. Organisms with efficient digestion absorb more isotopically enriched nutrients, resulting in higher tissue enrichment. Conversely, those with lower digestion efficiency retain more isotopically depleted components, leading to lower tissue enrichment. While dietary protein quality and quantity can influence these processes, their precise impact on Δ^15^N values remains unclear [75].

Unlike organisms consuming protein-rich diets [56], those on low-protein diets often catabolize their own tissues to meet nitrogen demands. This catabolism results in ^15^N enrichment through deamination and transamination [76, 77]. While it had been reported higher Δ^15^N values in congeneric crayfish species (*Orconectes rusticus* and *O. virilis*) [78], our *C. quadricarinatus* exhibited lower Δ^15^N values (1.4–1.5‰ in muscle and 0.5–1.1‰ in hepatopancreas), presenting a contrasting pattern.

Typically, Δ^15^N values decrease as dietary protein quality and quantity increase, with high Δ^15^N values indicating potential nutritional deficiencies (inadequate assimilation or unmet requirements) [79]. Our diets contained lower protein levels compared to previous studies [80], suggesting potentially higher Δ^15^N values. However, our observed low Δ^15^N and Δ^13^C values fall within the crustacean range [50, 78, 81, 82]. Further research is needed to identify the factors underlying these low Δ^15^N values in *C. quadricarinatus*.

The hepatopancreas, a metabolically active tissue, serves as the central metabolic organ and primary storage site in decapods [83]. It absorbs ingested food [84], synthesizes digestive enzymes, and stores energy and nutrients as glycogen and lipids within R-cells [85]. Lipids constitute the primary energy reserve in adult *C. destructor* hepatopancreas, readily mobilizing as needed [86]. Lipid levels in *C. quadricarinatus* hepatopancreas significantly declined during 15-day starvation, particularly on days 12 and 15, indicating lipolysis for energy provision [87]. Lipid mobilization is influenced by factors such as lifespan, habitat (temperature, current, and nutrient availability), and molting rate, with slower molting correlating to lower metabolic rates and reduced macromolecule mobilization during starvation.

Ammonia, a byproduct of amino acid breakdown, is the primary excretion product in most crustaceans [88]. Tissues with high protein turnover and degradation, like the hepatopancreas, exhibit elevated ^15^N enrichment compared to growing tissues such as muscle due to nitrogen isotope fractionation during amino acid deamination and transamination [89]. Ammonia's depleted ^15^N content enriches body tissues, especially under protein-poor diets with insufficient metabolic energy [90]. Notably, dietary regimes influence isotope fractionation, as seen in juvenile blue crabs *Callinectes sapidus* [56].

Isotopic value assimilation from dietary resources depends on growth rates, involving new tissue formation and material replacement due to catabolic turnover [91]. Fantle et al. [56] highlighted differences in essential and nonessential amino acid pathways in juvenile blue crabs, with essential amino acids undergoing less isotopic fractionation (more closely resembling dietary isotope profiles) than nonessential ones. The observed fractionation in crayfish juveniles thus reflects a delicate balance between dietary protein level and amino acid composition.

Stable isotopes and TDFs are crucial for studying aquatic organism trophic ecology and ecosystem function [30, 92, 93]. However, isotope fractionation varies widely based on species, tissue, diet, temperature, and other factors [30, 31, 81, 94, 95]. Consequently, researchers often rely on generalist TDF values (−0.6 to 2.7‰ for δ^13^C and 3 to 5‰ for δ^15^N) [22, 23, 90, 92] due to limited specific data. Generalist TDF values often diverge from experimentally derived data, potentially leading to inconsistencies when comparing dietary regimes from gut content and stable isotope analyses, as seen in *C. quadricarinatus* [96, 97] and *Metapenaeus macleayi* juveniles [98].

While mollusks and crustaceans generally exhibit lower δ^15^N enrichment [81], significant interindividual isotopic variation can occur even under controlled conditions. It has been suggested that variations in *C. destructor* juveniles stemmed from individual-specific enzyme regulation and diet type (single-source vs. mixed) [36]. This variability can influence fractionation estimates, leading to a wide range of values across similar species and tissues.

Our study revealed slightly higher fractionation in juveniles fed diet E, except for ^13^C in hepatopancreas. Muscle tissue exhibited average isotope fractionation estimates of + 4.9‰ for Δ^13^C and + 1.4‰ for Δ^15^N. While a meta-analysis reported average crustacean δ^15^N enrichment of 2‰ [81], aligning with our findings, notable deviations occur among decapods [78, 99, 100] and *Cherax* species (Table 4). These variations likely stem from factors including dietary quality, protein content [78, 79, 101, 102], tissue type, initial isotope values, dietary isotope values, and developmental stage [103, 104].

In addition to dietary factors, isotopic turnover and metabolic rates in crustaceans are influenced by a complex interplay of environmental (temperature, salinity, and oxygen level) and biological (life stage, size, sex, and physiological stage) factors. Temperature, a key environmental factor, significantly affects these rates. As temperature rises, metabolic processes accelerate, leading to faster turnover of carbon and nitrogen isotopes as a result of increased enzymatic activity and the acceleration of biochemical reactions involved in nutrient uptake, assimilation, and excretion [105]. Regarding potential mechanisms that could explain the observed differences in isotopic turnover rates and metabolic rates between the control and enzyme-supplemented diets, one hypothesis is that enzyme supplementation could reduce the energy expenditure on endogenous enzyme production, allowing a more efficient digestion and nutrient assimilation. Another possible mechanism is that the exogenous enzymes might work synergistically with the endogenous enzymes, increasing the rate of nutrient breakdown and absorption. These hypotheses could be tested in future studies by measuring not only isotopic turnover but also digestive enzyme activity levels, nutrient assimilation efficiency, and overall metabolic rates under different dietary conditions. Understanding the effects of those factors on metabolic processes and isotopic dynamics is crucial for accurate interpretation of isotopic data and for studies investigating fractionation in crustaceans.

Hence, it is recommended to conduct trials to evaluate the benefits of enzyme supplements in other specific crustacean rearing systems such as different growing temperature, density as a “social” factor that modulates agonistic activity and feeding activity in ponds/tanks among other factors.

## 5. Conclusion

This study pioneers the explicit evaluation of isotopic incorporation from a multienzymatic extract (derived from *P. muelleri* waste) into *C. quadricarinatus* juvenile diets. It assesses isotopic integration rates and TDFs in the muscle and hepatopancreas. Adding the multienzymatic extract to the control diet significantly accelerated δ^13^C turnover in both tissues, reflecting improved digestion and assimilation. Notably, tissue-specific responses underscore the hepatopancreas' crucial metabolic role and molting's influence on isotopic patterns.

The present investigation provides the first isotope fractionation estimates and TDFs for the species, revealing higher fractionation in extract-fed juveniles. Our findings highlight the importance of using species-specific data rather than generalist TDF values in trophic ecology studies, as dietary quality and tissue type significantly influence these values. The research provides new insights into diet composition, stable isotope dynamics, physiological responses, and the interplay between endogenous and exogenous digestive enzymes in juvenile *C. quadricarinatus*.

## Figures and Tables

**Table 1:** Stepwise regression parameters for the mathematical conversion of stable isotopes (δ^13^C and δ^15^N) between bulk and lipid-free tissues (pleon muscle and hepatopancreas) of *Cherax quadricarinatus* juveniles fed on two different diets.

Tissue		Estimate	SE	*t*	*p*	Residual SE (n)	*R* ^2^
δ^13^C							
Pleon	Intercept	−3.646	1.520	−2.40	0.021	0.143 (38)	0.946
	*a*	0.976	1.035	24.06	<0.0001	—	—
	*b*	0.994	0.312	3.09	0.004	—	—
Hepatopancreas	Intercept	−4.930	0.969	−5.09	<0.0001	0.263 (38)	0.924
	*a*	0.683	0.040	17.25	<0.0001	—	—
	*b*	0.081	0.013	6.25	<0.0001	—	—

δ^15^N							
Pleon	Intercept	1.371	0.529	2.59	0.014	0.148 (39)	0.827
	*a*	0.858	0.064	13.35	<0.0001	—	—
	*b*	—	—	—	n.s.	—	—
Hepatopancreas	Intercept	—	—	—	n.s.	0.087 (39)	0.978
	*a*	1.003	0.006	175.450	<0.0001	—	—
	*b*	0.011	0.003	3.91	<0.001	—	—

*Note:* Equation model (*δX*_lipid-free_ = Intercept + *b* × *δX*_bulk_ + *c* × C:N_bulk_) modified from [62]. Fitting was performed with pooled data from both dietary treatments.

Abbreviations: C, control diet; E, enzyme extract; n.s., not significant; SE, standard error.

**Table 2:** Mean stable isotope (δ^13^C and δ^15^N) and C:N values in diets (C and E) and in the muscle and hepatopancreas of *Cherax quadricarinatus* juveniles (from day 0, initial, to day 90, final) (*n* = 3).

	δ^13^C (‰)	δ^15^N (‰)	Total C (%)	Total N (%)	C:N
Diet					
Lipid-free C	−25.4 ± 0.3	6.7 ± 0.6	40.8 ± 1.5	5.5 ± 0.3	7.4 ± 0.1
Lipid Free E	−25.0 ± 0.2	7.4 ± 0.4	39.2 ± 0.9	5.3 ± 0.1	7.4 ± 0.0
Bulk C	−26.8 ± 0.1	7.1 ± 0.3	44.9 ± 0.8	4.6 ± 0.1	9.7 ± 0.0
Bulk E	−26.8 ± 0.1	7.3 ± 0.1	43.3 ± 2.4	4.4 ± 0.3	9.7 ± 0.2
Juveniles, pleon*⁣*^*∗*^					
Initial	−20.4 ± 0.0	8.2 ± 0.1	38.7 ± 1.4	11.5 ± 0.5	3.4 ± 0.06
Final, C	−22.1 ± 0.1	8.4 ± 0.1	35.3 ± 3.4	10.9 ± 1.1	3.2 ± 0.01
Final, E	−21.8 ± 0.3	8.8 ± 0.2	38.7 ± 3.6	11.9 ± 1.1	3.2 ± 0.03
Juveniles, hepatopancreas*⁣*^*∗*^					
Initial	−20.8 ± 0.1	6.4 ± 0.1	54.9 ± 2.8	7.7 ± 0.3	7.1 ± 0.4
Final, C	−23.3 ± 0.1	7.6 ± 0.1	60.2 ± 1.7	5.3 ± 1.2	11.8 ± 2.8
Final, E	−23.0 ± 0.3	8.3 ± 0.3	60.1 ± 1.3	4.9 ± 0.5	12.3 ± 1.4

*Note*: Difference *δX* = *δX*_final_ − *δX*_initial_.

*⁣* ^*∗*^Lipid-free tissue for δ^13^C and δ^15^N.

**Table 3:** Parameter estimates for best fit models predicting δ^13^C and δ^15^N values in the hepatopancreas and muscle of *Cherax quadricarinatus* juveniles.

			δY_eq_	SE δY_eq_	*m*	*R* ^2^	D_50_	D_95_	G_50_	G_95_	Δδ	Δδ'
δ^13^C	Pleon	Diet C	−22.07	0.05	0.077	0.898	7.9	34.2	1.13	1.68	4.7	3.3
		Diet E	−21.59	0.09	0.153	0.605	4.3	18.5	1.03	1.39	5.1	3.4
	Hepatopancreas	Diet C	−23.37	0.05	0.192	0.967	3.4	14.8	1.03	1.30	3.4	2.0
		Diet E	−23.26	0.08	0.304	0.907	2.2	9.6	1.01	1.15	3.5	1.7

δ^15^N	Pleon	Diet C	8.46	0.14	0.033	0.131	15.8	68.3	1.33	1.35	1.4	1.8
		Diet E	8.75	0.32	0.020	0.268	23.4	101.2	1.51	5.11	1.6	1.3
	Hepatopancreas	Diet C	7.59	0.14	0.023	0.786	20.4	88.2	1.45	1.46	0.5	0.9
		Diet E	8.33	0.25	0.025	0.800	20.2	87.3	1.44	3.04	1.1	0.9

*Note:* Metabolic turnover rate: *m* (% day^−1^). Turnover rates: D_50_, D_95_ (days) and G_50_, G_95_ (x-fold increase in biomass). Δδ': tissue–diet discrimination factors with lipid-free diets.

Abbreviations: C, control diet; E, enzyme extract diet.

**Table 4:** Model parameters for isotopic incorporation rate models predicted for *Cherax* species.

	Species	Size (cm)	Tissue	Half-life (days)	Equilibrium (days)	Discrimination factor Δ (‰)
δ^13^C	*C. destructor* ^a^	7–10	M	—	180	−1.1
	—	—	F	—	—	—
	*C. destructor* ^b^	—	M	4.3–7.9	—	−1.36–2.11
	*—*	—	C	—	—	—
	*C. destructor* ^c^	2.2	M	—	—	1.57*⁣*^*∗*^
	*C. destructor* ^d^	—	M	—	—	2.64–2.80
	*C. quadricarinatus* ^e^	4.3	M	4.3–7.9	18.5–34.2	4.7–5.2
	*—*	—	H	2.2–3.4	9.6–14.8	3.4–3.5
	—	—	M	—	—	3.3–3.4*⁣*^*∗∗*^
	—	—	H	—	—	1.7–2.0*⁣*^*∗∗*^

δ^15^N	*C. destructor* ^a^	7–10	M	19	80	1.5
	—	—	F	—	—	0.9
	*C. destructor* ^b^	—	M	—	—	0.12–3.74
	*—*	—	C	—	—	—
	*C. destructor* ^c^	2.2	M	—	—	3.3*⁣*^*∗*^
	*C. destructor* ^d^	—	M	—	—	5.33–5.83
	*C. quadricarinatus* ^e^	4.3	M	15.8–23.4	68.3–101.2	1.4–1.5
	—	—	H	20.2–20.4	87.3–88.2	0.5–1.1
	—	—	M	—	—	1.3–1.7*⁣*^*∗∗*^
	—	—	H	—	—	0.9*⁣*^*∗∗*^

Abbreviations: Δ (‰), diet–tissue discrimination factor; C, carapace; F, feces; H, hepatopancreas; M, muscle.

^a^Carolan et al. [36].

^b^Mazumder et al. [38].

^c^Mazumder et al. [37].

^d^Duffy et al. [35].

^e^Present study.

*⁣* ^*∗*^Diet normalized for lipids; *⁣*^*∗∗*^ lipid-free diet.

## Data Availability

The data are available upon request to the corresponding author.
